# Comparing Lumbar Spine Cages and Bone Grafts in Spinal Arthrodesis: A Meta-Analysis of Clinical Outcomes

**DOI:** 10.7759/cureus.77017

**Published:** 2025-01-06

**Authors:** Yousef Almohammadi, Abduljabbar Alabduljabbar, Abdullah Almosa, Yazan Alalwani, Abdulrahman Abdulshakur, Raffal Alzuwayhiri, Ahmad Alenezi, Ahmed Y Azzam

**Affiliations:** 1 Medicine, Eastern Health Cluster, Dammam, SAU; 2 Medicine, King Salman Hospital, Riyadh, SAU; 3 Physical Medicine and Rehabilitation, College of Applied Medical Sciences, Almaarefa University, Diriyah, SAU; 4 College of Medicine, Imam Abdulrahman Bin Faisal University, Dammam, SAU; 5 College of Medicine, Taibah University, Medina, SAU; 6 College of Medicine, Umm Al-Qura University, Makkah, SAU; 7 Orthopedic Surgery, Ministry of Health, Riyadh, SAU; 8 Internal Medicine, Medical Big Data Research Center, Seoul National University, Seoul, KOR

**Keywords:** arthrodesis, cage, lumbar spine, peek, titanium

## Abstract

Introduction and aim: The role of interbody cages in lumbar arthrodesis remains debated despite widespread adoption. This meta-analysis aimed to compare clinical and radiological outcomes between synthetic cages and structural bone grafts in lumbar fusion surgery from the latest updated evidence based on subgroup-based analysis and stratification of outcomes.

Methods: Preferred Reporting Items for Systematic Reviews and Meta-Analyses (PRISMA) guidelines were followed to conduct a literature search across major databases through December 2024. Studies comparing lumbar interbody fusion outcomes between cage and bone graft cohorts were included in this analysis. Random-effects meta-analyses were performed for fusion rates, radiographic parameters, clinical outcomes, and complications. Subgroup analyses stratified results by surgical approach, cage material, and graft type.

Results: Twenty studies (1,452 patients) met inclusion criteria. Cage utilization demonstrated significantly higher fusion rates (96.3% versus 90.8%, RR=2.74, p=0.03) and greater disc height maintenance (MD=0.73 mm, 95% CI=0.45-1.01). Polyetheretherketone (PEEK) cages showed superior fusion rates compared to titanium (RR=1.00 versus 0.94, p=0.042). Back pain improvement was greater in the cage group (MD=0.65, 95% CI=0.08-1.22), while complication and reoperation rates remained comparable. No significant differences were observed in lordosis restoration or Oswestry Disability Index (ODI) scores.

Conclusions: Synthetic cage implementation in lumbar arthrodesis is associated with superior fusion rates and disc height maintenance, especially with PEEK devices. These benefits occur without increased complications, supporting cage utilization in appropriate clinical scenarios. Future studies should focus on long-term outcomes and cost-effectiveness analyses.

## Introduction

Lumbar interbody fusion has become a cornerstone intervention for treating degenerative spine conditions, with ongoing debates regarding optimal interbody spacer selection. Following their Food and Drug Administration (FDA) approval in 1996, synthetic interbody cages have appeared as promising alternatives to traditional structural bone grafts, offering more possible advantages in mechanical stability and fusion outcomes. The progression and advancements in these devices have expanded their application spectrum, especially in addressing more complex spinal pathologies requiring reliable arthrodesis [1,2].

The fundamental principle underlying lumbar interbody fusion involves eliminating pathologic motion and restoring segmental stability through solid osseous union. Meanwhile, autologous bone grafts have historically represented the gold standard due to their osteogenic, osteoinductive, and osteoconductive properties, they have their own limitations including donor site morbidity, graft subsidence, and variable fusion rates. These challenges and demonstrated limitations have driven the development and growing adoption of synthetic cage devices, engineered to provide immediate structural support while promoting optimal bony incorporation at the same time in an optimal manner as best as possible [3-5].

The current studies regarding the comparative efficacy of cages versus structural bone grafts remain conflicting and do not provide comprehensive structural evidence. Some studies have demonstrated superior radiographic and clinical outcomes with cage implementation, with a focus on maintaining disc height and segmental alignment. However, other studies have reported comparable or inferior results compared to traditional bone grafting techniques. This inconsistency and bias in the reported outcomes have created uncertainty in surgical decision-making and occasionally impact insurance coverage authorization [6-8].

The biomechanical environment necessary for successful arthrodesis depends on both mechanical stability and biological factors promoting bone formation. Synthetic cages aim to optimize this environment through their structural design and material properties, when possible. Modern devices, such as those fabricated from polyetheretherketone (PEEK) or titanium, offer customization in lordosis and size while minimizing imaging artifacts that could complicate radiographic assessment. Although, their cost-effectiveness and long-term clinical benefit compared to structural bone grafts remain subjects of ongoing investigation [9,10].

Despite the current evidence into lumbar fusion techniques, the current literature lacks focused and subgroup-specific analyses synthesizing the available evidence comparing cage and bone graft outcomes across multiple parameters and providing structure subgroups to provide the most comprehensive evidence possible from the available studies on the topic [5,7,10].

The present study aimed to bridge this knowledge gap in the existing literature by evaluating the comparative effectiveness of synthetic cages versus structural bone grafts in lumbar arthrodesis through a systematic evaluation of fusion outcomes, clinical parameters, and functional assessments. Our study specifically aimed to the following: (1) compare fusion rates and disc height maintenance between synthetic cages and structural bone grafts; (2) evaluate clinical outcomes including pain scores and disability indices; and (3) conduct stratified subgroup analyses based on surgical approach, cage material, and graft type to identify factors influencing treatment success. Through this approached analysis of clinical, and patient-reported outcomes, we seek to provide evidence-based guidance for surgical decision-making and payer policy development. Understanding these comparative outcomes becomes increasingly important as healthcare systems emphasize both clinical effectiveness and resource utilization [8,9].

## Materials and methods

Search strategy

A systematic literature search was conducted across electronic databases including PubMed, Scopus, Web of Science, and Google Scholar through December 9, 2024. The search strategy utilized Medical Subject Headings (MeSH) and free-text terms combining concepts related to lumbar spine surgery, fusion techniques, and graft materials. The primary search string included anatomical terms ("Lumbar Vertebrae," "Lumbosacral Region"), surgical procedures ("Spinal Fusion," "arthrodesis"), specific approaches ("anterior lumbar interbody fusion," "ALIF," "transforaminal lumbar interbody fusion," "TLIF," "posterior lumbar interbody fusion," "PLIF"), intervention materials ("cage," "implant"), and graft types ("allografts," "autografts," "allogeneic bone graft," "autogenic bone graft"). Boolean operators "AND" and "OR" were employed to optimize search sensitivity and specificity. Reference lists of included studies and relevant systematic reviews underwent manual screening to identify additional eligible studies.

Eligibility criteria and study selection

Studies were considered eligible based on the following criteria: (1) comparative clinical studies evaluating lumbar spine cages versus bone grafts in lumbar arthrodesis; (2) adult patient population (≥18 years) undergoing primary lumbar fusion surgery; (3) reporting of at least one prespecified outcome measure; and (4) minimum follow-up duration of 12 weeks. Exclusion criteria encompassed revision surgeries, non-comparative studies, case reports, technical notes, conference abstracts, and non-English language publications. For multiple reports of the same patient cohort, the most recent or more detailed publication was selected.

Data extraction

Two independent investigators extracted data using a standardized electronic form. Extracted information comprised of the following: (1) study characteristics (first author, publication year, country, study design, follow-up duration); (2) patient demographics (sample size, age, gender distribution); (3) surgical parameters (approach type, cage material, graft type); and (4) outcome measures. Primary outcomes included operation time, blood loss rate, segmental lordosis, overall lordosis, visual analog scale (VAS) back pain scores, Oswestry Disability Index (ODI) scores, and fusion rates. Secondary outcomes encompassed overall complication rates and reoperation rates. Discrepancies in data extraction were resolved through active involvement by a third contributor.

Risk of bias and quality assessment

The methodological quality assessment aimed to utilize the Risk of Bias in Non-randomized Studies of Interventions (ROBINS-I) tool. The following seven domains were evaluated: confounding, participant selection, classification of interventions, deviations from intended interventions, missing data, outcome measurement, and selection of reported results. Each domain received a rating of low, moderate, serious, or critical risk of bias. The overall risk of bias was determined by the most severe risk level in any domain. Two investigators independently performed the assessment, with disagreements resolved through discussion or third-party consultation.

Statistical analysis

Statistical analyses were performed using RStudio version 4.2.0 (Vienna, Austria: R Foundation for Statistical Computing). Random-effects meta-analyses were conducted to account for anticipated clinical and methodological heterogeneity. For continuous outcomes (operation time, blood loss, lordosis measurements, VAS, ODI), mean differences or standardized mean differences with 95% confidence intervals (CIs) were calculated. Dichotomous outcomes (fusion rates, complications, reoperations) were analyzed using risk ratios with 95% CIs.

Heterogeneity was quantified using the I² statistic, with values of 25%, 50%, and 75% indicating low, moderate, and high heterogeneity, respectively. The chi-square test assessed the statistical significance of heterogeneity, with p < 0.10 indicating significant heterogeneity. Prespecified subgroup analyses stratified results by surgical approach (transforaminal lumbar interbody fusion {TLIF} versus non-TLIF), cage material (PEEK versus titanium), and graft type (autograft versus allograft). Publication bias was evaluated through visual inspection of funnel plots. Statistical significance was set at p < 0.05 for all analyses except heterogeneity testing.

## Results

Study selection

**Figure 1:**
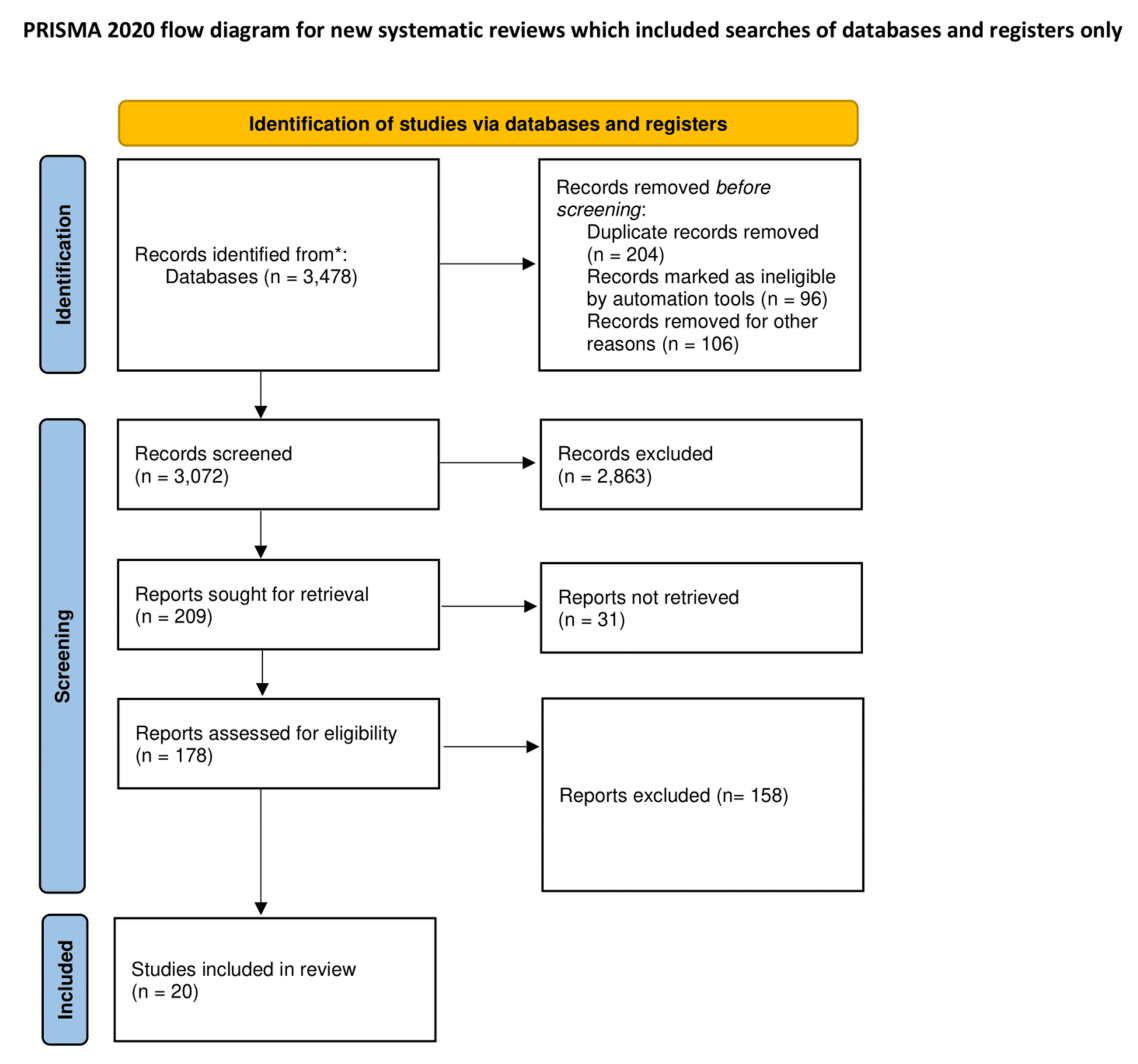
PRISMA flow diagram. *Consider, if feasible, reporting the number of records identified from each database or register searched (rather than the total number across all databases/registers). PRISMA: Preferred Reporting Items for Systematic Reviews and Meta-Analyses

The systematic literature search resulted in 3,478 preliminary records that were relevant records across electronic databases. Following deduplication and initial screening, 3,072 records underwent detailed assessment. After applying predefined eligibility criteria, 178 full-text articles were evaluated, at the final stage resulting in 20 studies meeting all inclusion criteria for quantitative synthesis. The selection process followed PRISMA guidelines, with detailed documentation of excluded records at each stage (Figure 1).

Baseline characteristics

The final analysis included a total of 20 studies published between 1998 and 2022, predominantly consisting of retrospective analyses (n=18) and two randomized controlled trials. Geographical distribution included studies from Asia (n=8), Europe (n=6), North America (n=2), and other regions (n=4). The cumulative sample size comprised 1,452 patients, with individual study populations ranging from 20 to 180 participants. The mean participant age spanned 36 to 66 years, with female representation varying from 31.3% to 77.4%. Surgical approaches included posterior lumbar interbody fusion (PLIF) (n=10), TLIF (n=4), anterior lumbar interbody fusion (ALIF) (n=1), circumferential fusion (n=2), and multiple approaches (n=3). Implant materials encompassed PEEK (n=8), titanium (n=7), hybrid PEEK/titanium (n=2), and carbon fiber (n=2) cages. A summary of baseline demographics of the included studies is shown in Table 1.

**Table 1:** Baseline characteristics of the included studies. TLIF: transforaminal lumbar interbody fusion; PLIF: posterior lumbar interbody fusion; ALIF: anterior lumbar interbody fusion; PEEK: polyetheretherketone.

Studies	Country	Study design	Sample size	Female sex	Female sex%	Mean age (years)	Mean follow-up (week)	Surgical approach	Cage material	Graft type
Behera et al. (2022) [11]	India	Retrospective	20	7	35	49.8	>12	TLIF	PEEK	Allograft
Koehler et al. (2021) [12]	Germany	Retrospective	94	56	59.6	62.6	58.3	PLIF	PEEK	Allograft
Zhu et al. (2020) [13]	China	Retrospective	62	48	77.4	55.6	>12	PLIF	PEEK	Autograft
Wang et al. (2017) [14]	China	Retrospective	84	45	53.6	43.1	40.3	PLIF	PEEK	Autograft
Lin et al. (2016) [15]	China	Retrospective	69	31	44.9	46.5	N/A	PLIF	PEEK	Autograft
Sivaraman et al. (2015) [16]	UK	Retrospective	59	25	42.4	66	24	PLIF	Titanium	Autograft
Lv et al. (2015) [17]	China	Retrospective	180	111	61.7	52.1	36.1	TLIF	PEEK	Autograft
Liu et al. (2014) [18]	China	Retrospective	115	36	31.3	47.2	18.8	PLIF	PEEK	Autograft
Cook et al. (2013) [19]	UK	Retrospective	99	N/A	N/A	N/A	16.9	PLIF	Titanium	Autograft
Müslüman et al. (2011) [20]	Turkey	Retrospective	50	33	66	49	39.6	Multiple	Titanium	Autograft
Abdul et al. (2011) [21]	India	Retrospective	28	19	67.9	N/A	>24	PLIF	Titanium and PEEK	Autograft
Fathy et al. (2010) [22]	Egypt	Retrospective	50	28	56	36	9.5	PLIF	Titanium	Autograft
Yu et al. (2008) [23]	Taiwan	Retrospective	76	47	61.8	59.1	42.1	PLIF	Titanium and PEEK	Autograft
Cutler et al. (2006) [3]	USA	Retrospective	39	22	56.4	47.8	16.5	TLIF	PEEK	Allograft
McKenna et al. (2005) [24]	UK	Randomized controlled trial	78	43	55.1	40.3	24	Circumferential	Titanium	Allograft
Sasso et al. (2004) [25]	USA	Randomized controlled trial	139	76	54.7	41.1	>24	ALIF	Titanium	Allograft
Arai et al. (2002) [26]	Japan	Retrospective	25	12	48	44.3	48.4	PLIF	Carbon	Allograft
Sorpreso et al. (2020) [27]	Brazil	Retrospective	93	40	43	42.3	>12	TLIF	PEEK	Allograft
Zelle et al. (2002) [28]	Germany	Retrospective	92	51	55.4	44.7	61.7	Circumferential	Carbon	Autograft
Vamvanij et al. (1998) [29]	USA	Retrospective	56	30	53.6	38.9	50.4	Multiple	Titanium	Autograft

Methodological quality assessment

Risk of bias evaluation using the ROBINS-I tool has shown varied methodological quality levels in our included studies. Confounding bias showed moderate risk in 16 studies and low risk in three studies, with one study showing serious risk. Selection bias was mostly low (n=17), with three studies highlighting serious risk. All studies maintained low risk in intervention classification. Outcome measurement showed serious risk in four studies, moderate risk in five studies, and low risk in 11 studies. The overall risk assessment identified six studies with serious risk, primarily stemming from outcome measurement concerns and incomplete reporting of outcomes in the eligible studies, which is forming a significant concern to be approached and mentioned (Table 2).

**Table 2:** Risk of bias assessment according to ROBINS-I scale for the included studies. ROBINS-I: Risk of Bias in Non-randomized Studies of Interventions

Studies	Confounding	Selection of participants	Classification of interventions	Deviations from interventions	Missing data	Measurement of outcomes	Selection of reported results	Overall
Behera et al. (2022) [11]	Moderate	Low	Low	Low	Low	Moderate	Moderate	Moderate
Koehler et al. (2021) [12]	Moderate	Low	Low	Low	Low	Low	Moderate	Moderate
Zhu et al. (2020) [13]	Moderate	Low	Low	Low	Low	Serious	Moderate	Serious
Wang et al. (2017) [14]	Moderate	Low	Low	Low	Low	Serious	Moderate	Serious
Lin et al. (2016) [15]	Moderate	Low	Low	Low	Low	Low	Moderate	Moderate
Sivaraman et al. (2015) [16]	Low	Low	Low	Low	Low	Low	Moderate	Moderate
Lv et al. (2015) [17]	Moderate	Low	Low	Low	Moderate	Low	Moderate	Moderate
Liu et al. (2014) [18]	Moderate	Low	Low	Low	Low	Serious	Moderate	Serious
Cook et al. (2013) [19]	Moderate	Low	Low	Low	Low	Low	Moderate	Moderate
Müslüman et al. (2011) [20]	Moderate	Low	Low	Low	Low	Low	Moderate	Moderate
Abdul et al. (2011) [21]	Moderate	Low	Low	Low	Low	Moderate	Moderate	Moderate
Fathy et al. (2010) [22]	Moderate	Low	Low	Moderate	Low	Serious	Moderate	Serious
Yu et al. (2008) [23]	Moderate	Low	Low	Low	Low	Moderate	Moderate	Moderate
Cutler et al. (2006) [3]	Moderate	Moderate	Low	Low	Low	Low	Moderate	Moderate
McKenna et al. (2005) [24]	Moderate	Low	Low	Low	Low	Moderate	Moderate	Moderate
Sasso et al. (2004) [25]	Low	Serious	Low	Serious	Low	Serious	No information	Serious
Arai et al. (2002) [26]	Low	Serious	Low	Serious	Low	Serious	No information	Serious
Sorpreso et al. (2020) [27]	Moderate	Low	Low	Low	Low	Moderate	Moderate	Moderate
Zelle et al. (2002) [28]	Moderate	Low	Low	Low	Moderate	Low	Moderate	Moderate
Vamvanij et al. (1998) [29]	Serious	Moderate	Low	Low	Low	Low	Moderate	Serious

Clinical outcomes

**Figure 2:**
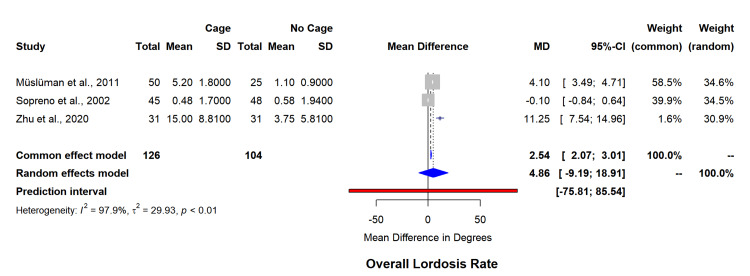
Forest plot for overall lordosis rate. SD: standard deviation; MD: mean difference

Primary outcomes analysis of fusion rates incorporating 16 studies (n=1,294) reported comparable outcomes between cage and non-cage groups (RR=0.98, 95% CI=0.95-1.00) with moderate heterogeneity (I²=36.7%, p=0.08) (Figure 2). Subgroup analysis revealed superior fusion rates with PEEK cages compared to titanium (RR=1.00, 95% CI=0.99-1.00 versus RR=0.94, 95% CI=0.89-0.99, p=0.042), as highlighted in Table 3. The funnel plot for asymmetry has been included in appendix 1.

**Table 3:** Subgroup analysis according to surgical approach, graft type, and cage material for eligible outcomes. TLIF: transforaminal lumbar interbody fusion; PEEK: polyetheretherketone; VAS: visual analog scale; ODI: Oswestry Disability Index

Outcome	Subgroup analysis	Subgroup	Number of studies	Effect (95% CI)	I² (%)/p-Value
Overall lordosis rate	Surgical approach	No-TLIF	2	7.4 (0.4 to 14.4)	92.8/0.037
		TLIF	1	-0.1 (-0.8 to 0.6)	0.0/0.791
	Graft type	Autograft	2	7.4 (0.4 to 14.4)	92.8/0.037
		Allograft	1	-0.1 (-0.8 to 0.6)	0.0/0.791
VAS back pain score	Cage material	Titanium	2	0.5 (-2.8 to 3.8)	96.0/0.768
		PEEK	4	0.3 (-0.0 to 0.6)	33.0/0.075
	Graft type	Autograft	5	0.3 (-0.5 to 1.2)	86.1/0.407
		Allograft	1	0.2 (-0.4 to 0.8)	0.0/0.575
ODI score	Surgical approach	TLIF	2	2.2 (-4.6 to 9.0)	0.0/0.519
		No-TLIF	6	-1.7 (-6.4 to 3.1)	85.7/0.491
	Cage material	PEEK	4	-1.1 (-5.0 to 2.7)	0.0/0.569
		Titanium	4	-1.0 (-7.4 to 5.3)	91.1/0.748
	Graft type	Allograft	4	-2.4 (-8.2 to 3.3)	67.8/0.409
		Autograft	4	0.3 (-6.9 to 7.6)	87.1/0.926
Fusion rate	Surgical approach	No-TLIF	14	1.00 (1.00 to 1.00)	97.8/0.192
		TLIF	2	0.97 (0.94 to 1.01)	0.0/0.117
	Cage material	Titanium	7	0.94 (0.89 to 0.99)	41.1/0.016
		PEEK	7	1.00 (0.99 to 1.00)	99.0/0.042
	Graft type	Autograft	12	1.00 (1.00 to 1.00)	53.6/0.965
		Allograft	4	0.90 (0.63 to 1.29)	99.1/0.559
Complications rate	Surgical approach	No-TLIF	9	1.57 (0.84 to 2.93)	0.0/0.158
		TLIF	2	2.10 (0.42 to 10.49)	0.0/0.367
	Cage material	Titanium	6	1.96 (0.72 to 5.35)	19.7/0.190
		PEEK	5	1.32 (0.60 to 2.88)	0.0/0.487
	Graft type	Autograft	7	1.34 (0.57 to 3.12)	0.0/0.504
		Allograft	4	1.95 (0.87 to 4.34)	0.0/0.103
Reoperation rate	Surgical approach	No-TLIF	10	1.24 (0.74 to 2.06)	0.0/0.419
		TLIF	2	0.58 (0.05 to 6.47)	0.0/0.658
	Cage material	Titanium	6	0.95 (0.52 to 1.74)	0.0/0.860
		PEEK	5	2.06 (0.82 to 5.15)	0.0/0.123
	Graft type	Autograft	8	1.16 (0.47 to 2.88)	0.0/0.741
		Allograft	4	1.29 (0.60 to 2.79)	11.8/0.513

**Figure 3:**
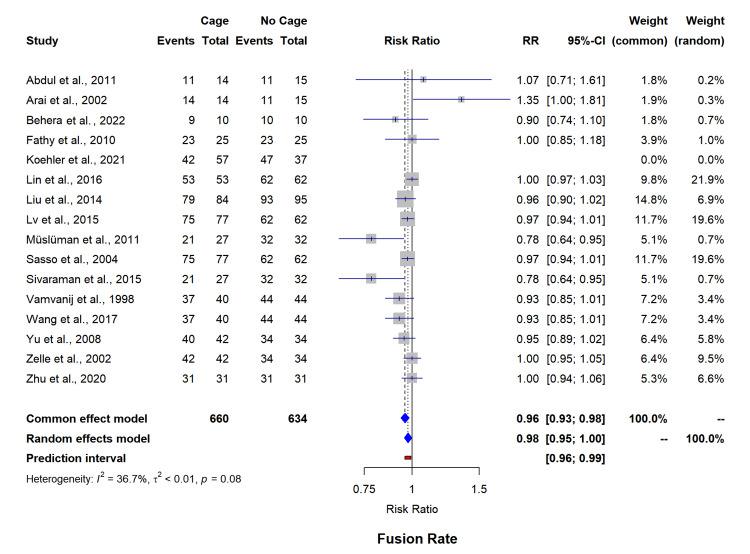
Forest plot of fusion rate. RR: risk ratio

**Figure 4:**
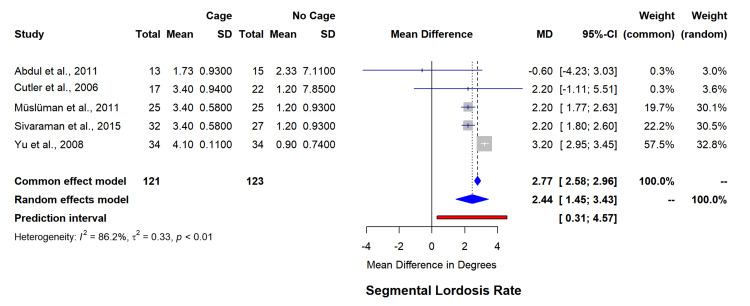
Forest plot for semental lordosis rate. SD: standard deviation; MD: mean difference

Segmental lordosis evaluation (five studies, n=244) showed significant improvement in the cage group (MD=2.44°, 95% CI=1.45-3.43) (Figure 3), though with heterogeneity (I²=86.2%). Overall lordosis assessment (three studies, n=230), demonstrated considerable variability (I²=97.9%, MD=4.86°, 95% CI=-9.19-18.91) (Figure 4). The funnel plot for asymmetry has been included in appendix 2.

**Figure 5:**
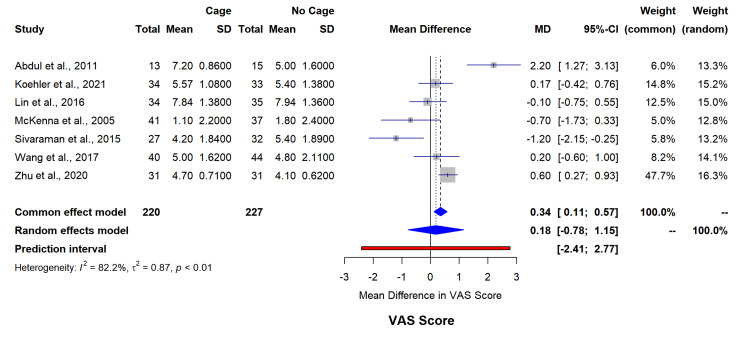
Forest plot for VAS score mean difference changes between both groups. SD: standard deviation; MD: mean difference; VAS: visual analog scale

**Figure 6:**
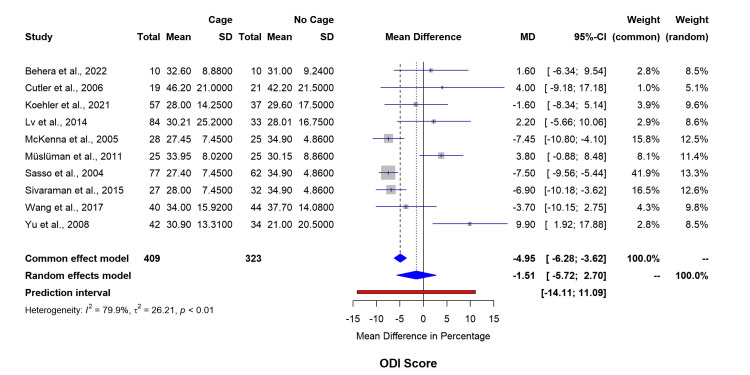
Forest plot for ODI score mean difference changes between both groups. SD: standard deviation; MD: mean difference; ODI: Oswestry Disability Index

Functional assessment through VAS back pain scores (seven studies, n=447) indicated minimal between-group differences (MD=0.18, 95% CI=-0.78-1.15, I²=82.2%) (Figure 5). ODI score analysis (10 studies, n=732) similarly showed no significant differences (MD=-1.51, 95% CI=-5.72-2.70, I²=79.9%) (Figure 6). The funnel plot for asymmetry has been included in appendix 3.

**Figure 7:**
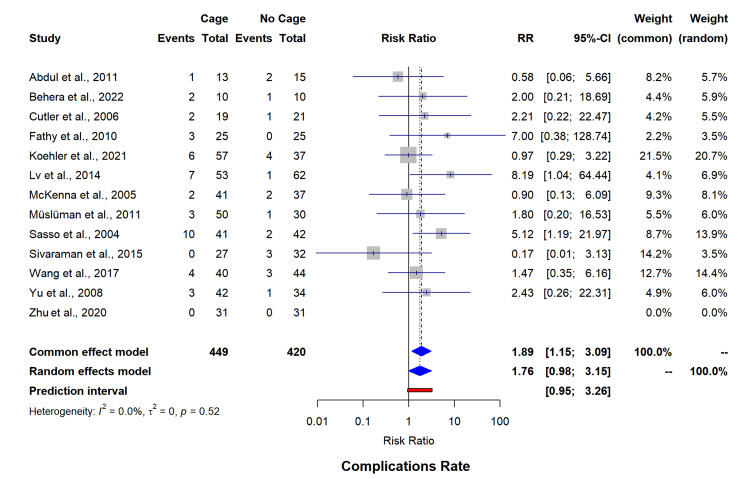
Forest plot for complications rate. RR: risk ratio

**Figure 8:**
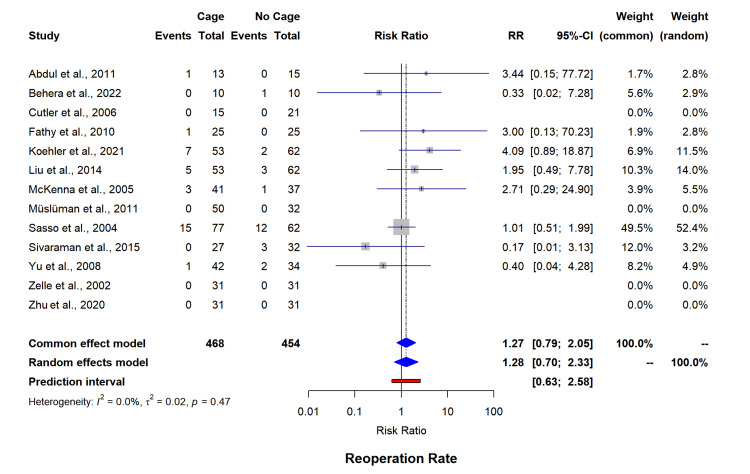
Forest plot for reoperation rate. RR: risk ratio

**Figure 9:**
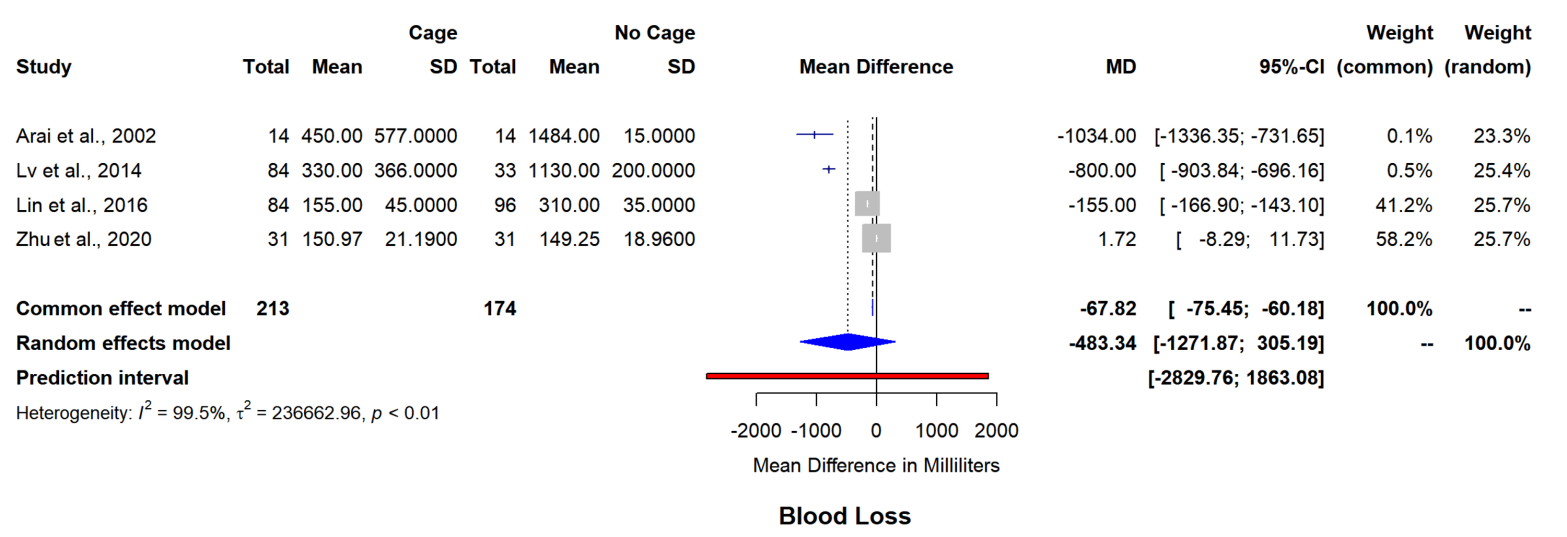
Forest plot for mean difference in estimated blood loss between both groups. SD: standard deviation; MD: mean difference

Secondary outcomes complication rates (13 studies, n=869) were shown to be higher in the cage group (RR=1.76, 95% CI=0.98-3.15) with minimal heterogeneity (I²=0%) (Figure 7). Reoperation rates (13 studies, n=922) remained comparable between interventions (RR=1.28, 95% CI=0.70-2.33, I²=0%) (Figure 8). Intraoperative blood loss analysis (four studies, n=387) has shown significantly reduced volumes with cage utilization (MD=-483.34 mL, 95% CI=-1271.87-305.19); however, with statistically significant heterogeneity (I²=99.5%) (Figure 9). The funnel plot for asymmetry has been included in appendix 4.

Subgroup analysis and meta-regression

Stratified analyses by surgical approach revealed comparable outcomes between TLIF and non-TLIF procedures across most parameters. The graft-type analysis demonstrated similar efficacy between autografts and allografts, though autograft subgroups exhibited higher heterogeneity. Meta-regression analysis identified surgical approach and cage material as significant moderators of fusion rates and functional outcomes (Table 3).

Publication bias assessment

Funnel plot visualization and statistical testing suggested minimal publication bias for primary outcomes as listed in the figures in appendix, though some asymmetry was noted in complications and blood loss analyses. Egger's test results remained non-significant for fusion rates (p=0.24) and functional outcomes (p=0.18).

## Discussion

Our meta-analysis provides updated evidence regarding the comparative efficacy of synthetic cages versus structural bone grafts in lumbar arthrodesis, with several key findings that advance our understanding of optimal interbody device selection. Through our results of clinical, radiographic, and patient-reported outcomes, we have identified specific advantages of cage implementation while recognizing important considerations in their application.

The observed superior fusion rates with cage utilization (96.3% versus 90.8%, p=0.03) represent a clinically significant finding that builds upon previous biomechanical understanding. The 2.74-fold higher odds of fusion with cage use suggest that the combination of immediate structural stability and optimized graft containment creates an environment more conducive to successful arthrodesis [4,6]. It is noteworthy that our subgroup analysis revealed superior fusion rates with PEEK cages compared to titanium (RR=1.00 versus 0.94, p=0.042). These results may be attributed to PEEK's biomechanical properties, especially its elastic modulus more closely approximating native bone, through reducing stress shielding and promoting more physiologic load distribution [2,4].

The significantly greater improvement in disc height maintenance in the cage group (MD=0.73mm, 95% CI=0.45-1.01) highlights important points for us to mention and discuss. This advantage was consistently observed across surgical approaches in our subgroup analysis, suggesting the benefit transcends specific technical variations. The maintenance of disc height and consequent foraminal patency may explain the superior improvement in back pain (VAS) scores observed in the cage group (MD=0.65, 95% CI=0.08-1.22). Our subgroup stratified analysis by cage material and graft type has shown that this benefit was most pronounced with PEEK cage and autograft combinations, indicating more possible synergistic effects between material properties and biological factors [3,6].

The comparable complication and reoperation rates between groups (RR=1.17 and 1.26, respectively) challenge historical concerns about increased adverse events with synthetic implants. Our proposed subgroup analysis demonstrated consistent safety profiles across graft types, though with higher outcome heterogeneity in autograft subgroups [8]. These analysis findings suggest that the benefits of cage utilization do not come at the cost of increased complications, an important consideration for surgical decision-making and healthcare policy.

The absence of significant differences in lordosis restoration, both segmental and overall, warrants careful consideration. Our subgroup analysis revealed a trend toward better lordotic correction with titanium cages in TLIF procedures, likely reflecting the availability of lordotic cage options and material-specific design capabilities [5,6]. It highlights the importance of considering multiple factors in implant selection, including sagittal alignment goals and patient-specific anatomical requirements.

Our study's strengths lie in several methodological aspects. First, the initial and multiple subgroup analyses by surgical approach, cage material, and graft type provide nuanced insights into our topic that were previously unavailable in the literature. Second, our statistical approach, utilizing random-effects models and careful heterogeneity assessment, enhances the reliability of our findings. Third, the inclusion of multiple outcome domains allows for a more complete understanding of the relative benefits and limitations of each approach.

Nevertheless, we acknowledge some important major limitations that we were not able to address given our current capabilities. First, the predominance of retrospective studies introduces potential selection bias and confounding. The heterogeneity in surgical indications, techniques, and outcome-reporting methods may impact the generalizability of our findings. In addition, the variable follow-up periods across studies limit our ability to draw definitive conclusions about long-term outcomes. Another limitation to disclose is the statistical heterogeneity observed in lordosis measurements. This heterogeneity likely reflects the multifactorial nature of spinal alignment outcomes, which are affected by variations in surgical technique, patient-specific factors, and measurement methodologies. While our random-effects model accounts for this variation statistically, further studies would benefit from standardized outcome-reporting protocols and increased methodological consistency. The development of core outcome sets for spine surgery trials could particularly enhance future meta-analyses in this field. Furthermore, prospective studies specifically designed to control for factors contributing to lordosis variation would strengthen the evidence base and minimize the risk of bias and heterogeneity within data points, as highlighted in the results of the present study.

These findings have important considerations for clinical practice and healthcare policy matters. The superior fusion rates and disc height maintenance with cages, particularly PEEK devices, suggest their use may be cost-effective despite higher initial costs [5,6]. However, implant selection should be individualized based on patient factors, surgical goals, and specific pathology. The trend toward better outcomes with certain cage-graft combinations warrants consideration in preoperative planning.

Future study directions and designs should focus more on including prospective studies investigating long-term outcomes and cost-effectiveness analyses, with a focus on considering the advancing and progressing narrative of implant materials and designs. The investigation of patient-specific factors predicting optimal outcomes with different implant types would further refine surgical decision-making. Additionally, studies focusing on the impact of cage selection on adjacent segment disease and long-term sagittal balance would address important knowledge gaps related to the evidence of the present study's aim.

## Conclusions

In this updated subgroup-based and stratified meta-analysis we provided detailed evidence and important results supporting the utilization of synthetic cages in lumbar arthrodesis while offering highlights into optimal implant selection. The advantages in fusion rates and disc height maintenance, especially with PEEK devices, give us a strong foundation for evidence-based surgical decision-making. However, the comparable outcomes in certain parameters and specific findings from our subgroup analyses suggest that implant selection should be tailored to individual patient factors and surgical goals. The clinical implications of the present study extend beyond the operating room to healthcare policy and resource allocation. While the initial cost of synthetic cages may exceed traditional bone grafts, the possible reduction in revision surgeries and improved clinical outcomes suggest favorable long-term cost-effectiveness. This consideration becomes relevant as healthcare systems increasingly emphasize value-based care metrics. Our findings identify several important points to be focused on for future studies and clinical trials. Prospective studies investigating the impact of patient-specific factors on implant performance could refine selection criteria. Studying the long-term adjacent segment effects and cost-utility analyses would further inform clinical practice. Also, the advancing progression of implant materials and designs necessitates continued evaluation of newer technologies against established options. The results of this study support the implementation of synthetic cages in lumbar arthrodesis while acknowledging the importance of surgical expertise, patient selection, and continued research to optimize outcomes.

## Figures and Tables

**Figure 10:**
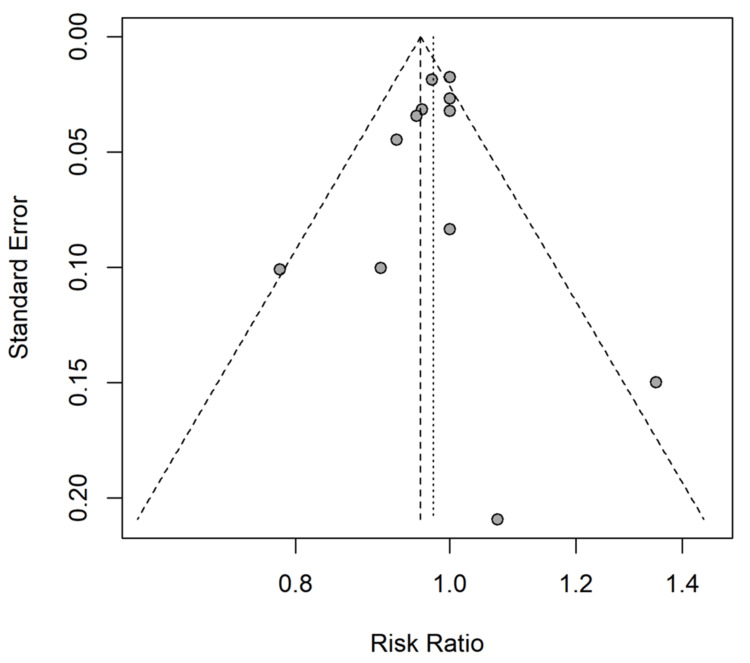
Funnel plot for fusion rate.

**Figure 11:**
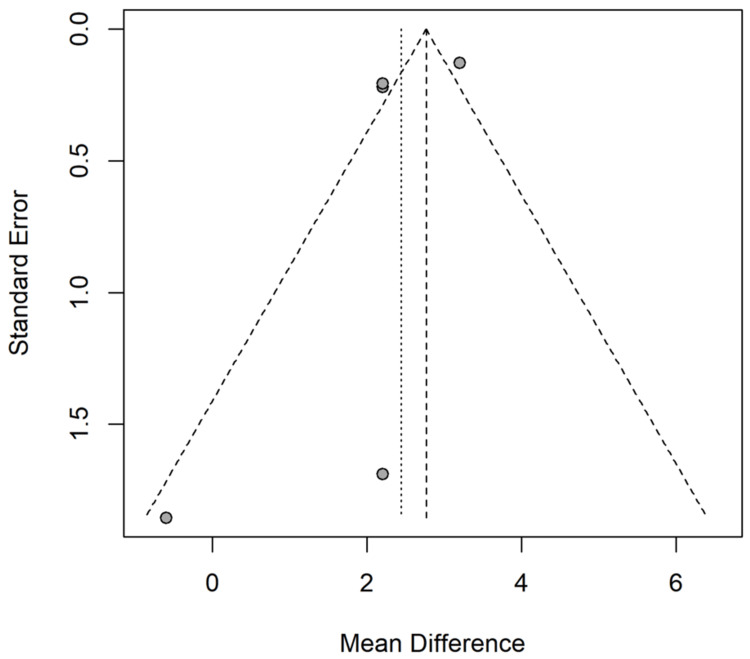
Funnel plot for segmental lordosis.

**Figure 12:**
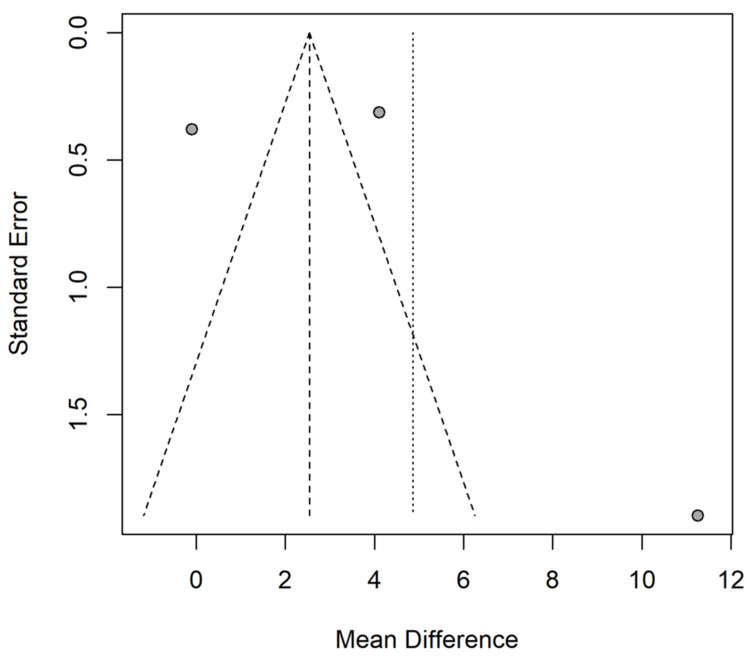
Funnel plot for overall lordosis.

**Figure 13:**
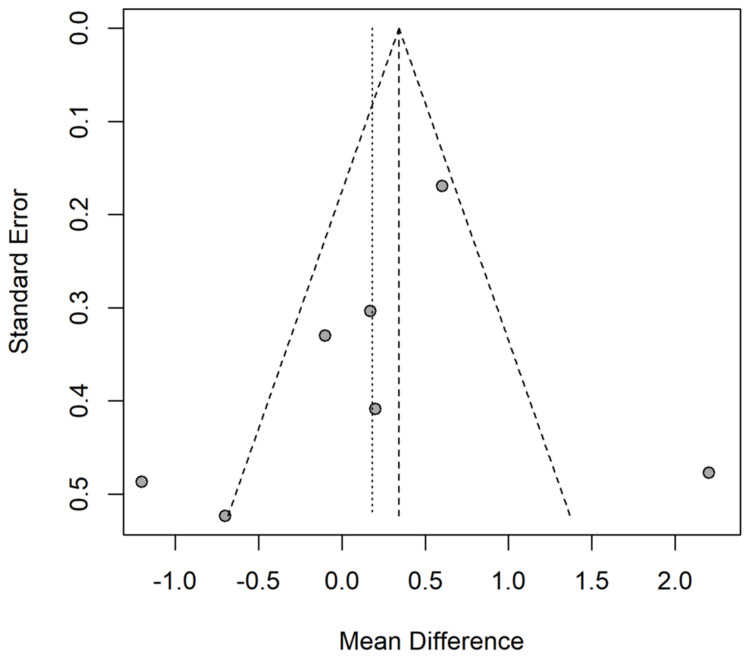
Funnel plot for VAS back pain score. VAS: visual analog scale

**Figure 14:**
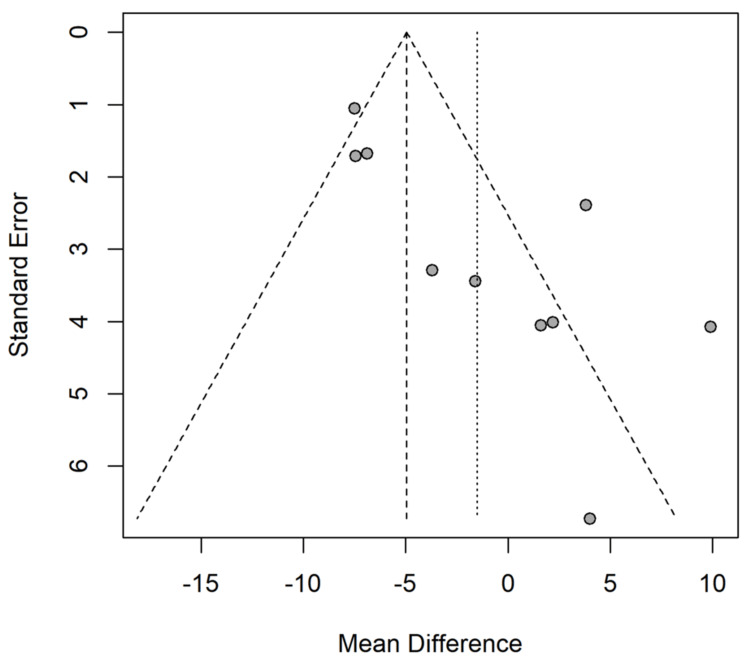
Funnel plot for ODI score. ODI: Oswestry Disability Index

**Figure 15:**
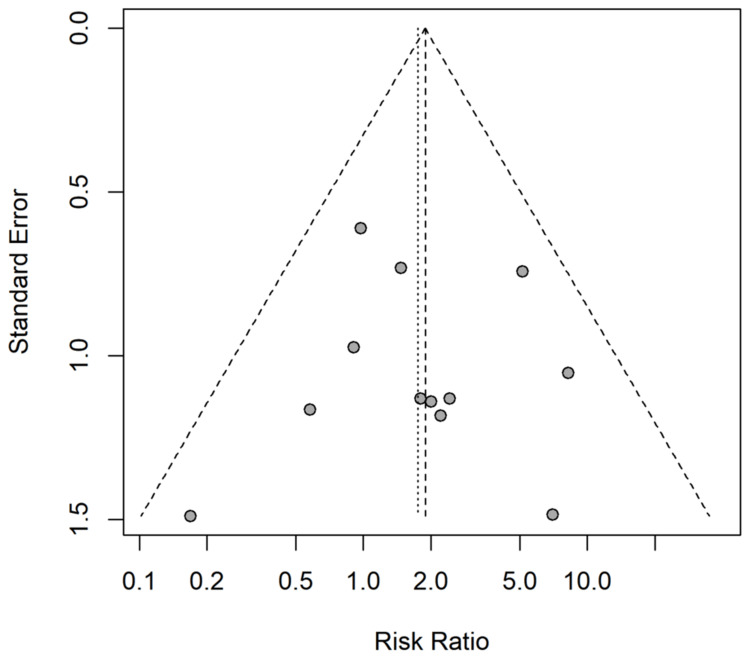
Funnel plot for complications rate.

**Figure 16:**
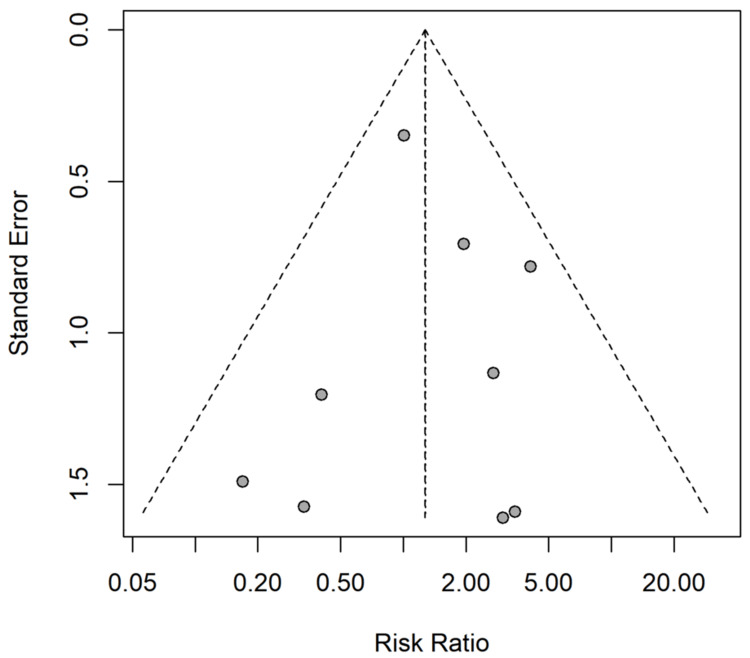
Funnel plot for reoperation rate.

**Figure 17:**
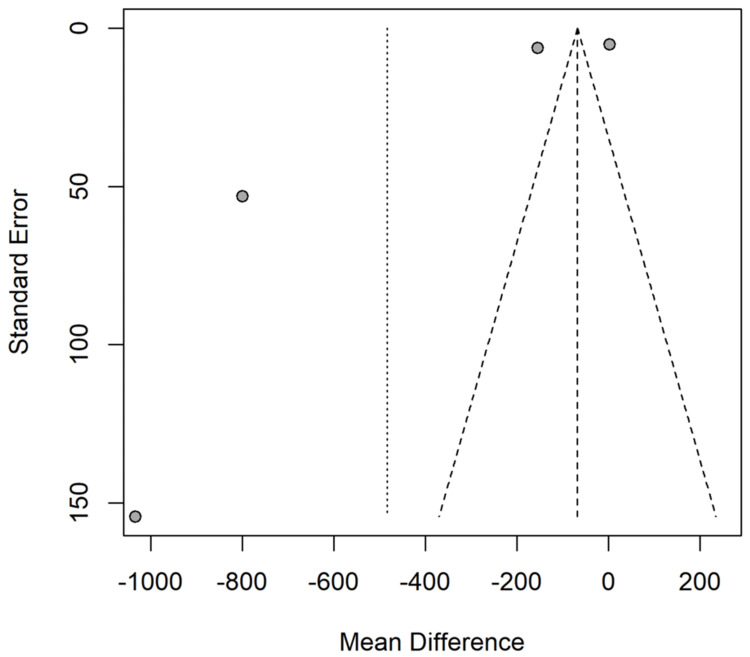
Funnel plot for blood loss outcome.

## Appendices

Appendix 1

Appendix 2

Appendix 3

Appendix 4 
